# Convolution-based approach for modeling the paliperidone extended release and Long-Acting Injectable (LAI) PK of once-, and three-monthly products administration and for optimizing the development of new LAI products

**DOI:** 10.1007/s10928-022-09835-7

**Published:** 2022-12-09

**Authors:** Roberto Gomeni, Françoise Bressolle-Gomeni

**Affiliations:** Pharmacometrica, Lieu-dit Longcol, 12270 La Fouillade, France

**Keywords:** Convolution-based model, Paliperidone, LAI, MIDD

## Abstract

The aim of this paper was to develop a convolution-based modeling approach for describing the paliperidone PK resulting from the administration of extended-release once-a-day oral dose, and once- and three monthly long-acting injectable products and to compare the performances of this approach to the traditional modeling strategy. The results of the analyses indicated that the traditional and convolution-based models showed comparable performances in the characterization of the paliperidone PK. However, the convolution-based approach showed several appealing features that justify the choice of this modeling as a preferred tool for modeling Long Acting Injectable (LAI) products and for deploying an effective model-informed drug development process. In particular, the convolution-based modeling can (a) facilitate the development of in vitro/in vivo correlation, (b) be used to identify formulations with optimal in vivo release properties, and (c) be used for optimizing the clinical benefit of a treatment by supporting the implementation of integrated models connecting in vitro and in vivo drug release, in vivo drug release to PK, and PK to PD and biomarker endpoints. A case study was presented to illustrate the benefits and the flexibility of the convolution-based modeling outcomes. The model was used to evaluate the in vivo drug release properties associated with a hypothetical once-a-year administration of a LAI product with the assumption that the expected paliperidone exposure during a 3-year treatment overlays the exposure expected after repeated administrations of a 3-month LAI product.

**Supplementary Information:**

The online version contains supplementary material available at 10.1007/s10928-022-09835-7.

## Introduction

Paliperidone is the major active metabolite of risperidone currently approved for the treatment of schizophrenia. A once-daily extended-release formulation (ER) INVEGA® was developed using an oral osmotic pump technology. Later, two long-acting injectable (LAI) paliperidone palmitate formulations were developed and approved for a once-monthly (PP1M: INVEGA® SUSTENNA®), once-every-3-months injection intervals (PP3M: INVEGA® TRINZA™), and, recently, once-every-6 months injection interval (PP6M: INVEGA HAFYERA™). All the LAI products were subcutaneously injected.

Paliperidone LAI products have unique pharmacokinetic properties characterized by a rate of drug absorption that is slower than their rate of elimination; hence, they exhibit flip-flop kinetics. Therefore, the shape of the terminal phase of the pharmacokinetic profile of these products reflects the rate of absorption, rather than the rate of elimination, as is usually referred as flip-flop PK [1].

The recommended dosage regimens for the LAI products, the management of missed doses, the switching strategy to formulations (oral to PP1M, PP1M to PP3M, and PP3M to PP6M) were based on model-based simulations conducted using population PK models. For this reason, the quality and the reliability of the population PK model plays a central role for supporting regulatory decision [2, 3], and for informing the appropriate patents used to protect the methodology for dose selection and for handling missed doses [4, 5].

In this framework, the structure and the predictive performance of the PK models are instrumental for qualifying the simulation results and for justifying the proposed dosing strategies, considering that different models can lead to different recommended dosing and switching strategies.

Historically, different structural models (referred as ‘traditional approach’) were developed to characterize the time-course of the absorption-rate-limited paliperidone PK for the ER and LAI products (PP1M and PP3M) using a conventional compartmental modeling approach. To date no structural model describing the population PK of PP6M has been published.

Recently, the convolution-based modeling approach was proposed as a powerful and flexible tool for modeling complex absorption pharmacokinetics of ER and LAI products, and for maximizing the benefit-risk ratio of a treatment [6].

Using this approach, the time course of the drug concentration can be described by convolving an input function with a disposition and elimination function when input and disposition functions are described by parametric models. This methodology provides a tool for developing an integrated modeling approach for describing the PK of the different LAI products using a common structural model for absorption and disposition but with a parameter-specific characterization of the drug absorption process. One of the benefits of this modeling approach is the possibility to dispose of a modeling framework for optimizing the development of alternative LAI products by identify the in vivo input function regulating the drug release suitable for delivering the expected exposure level at selected times.

The objectives of the present paper were: (a) to implement a convolution-based model for paliperidone ER and LAI products for describing the paliperidone PK resulting from the ER, PP1M, and PP3M product administrations, (b) to compare the performance of the traditional and convolution-based models, (c) to show how the convolution-based modeling approach is instrumental for supporting a model-informed drug development (MIDD) by evaluating the feasibility and the characteristics of a possible new paliperidone LAI once-a-year product.

## Methods

### Data

The paliperidone ER PK data were generated in a dose proportionality, open-label, randomized, 5-treatment, 5-period crossover study in 45 healthy males at dose levels of 3 mg, 6 mg, 9 mg, 12 mg, and 15 mg. The mean PK data were extracted from the European Medicines Agency scientific discussion document and used for model development [7].

The paliperidone PP1M PK data were generated in a single-dose, open label, randomized, parallel group study designed to evaluate the dose proportionality of 4 fixed doses of paliperidone: 25 mg, 50 mg, 100 mg, and 150 mg [8]. A total of 201 patients with schizophrenia were randomized to receive a single paliperidone injection in either the deltoid or the gluteal muscle. The mean data associated with the injections in the deltoid muscle at the different doses were used in the analysis.

The paliperidone PP3M PK single-dose data were generated in an open-label, randomized, phase I study designed to evaluate the PK, safety, and tolerability of PP3M following an intramuscular single dose injection in the gluteal or deltoid muscle of schizophrenic patients of the doses of 175 mg, 300 mg, 450 mg, and 525 mg [9]. The mean data associated with the injections in the deltoid muscle at the different doses were used in the analysis. The paliperidone PP3M PK repeated-dose data were generated in a double-blind, randomized, active-controlled, parallel-group multicenter clinical trial in schizophrenic patients. In this trial, PP3M was administered at the doses of 350 mg, and 525 mg. The study was organized in 3 phases: (1) a screening phase (up to 28 days), (2) an open-label maintenance phase (duration of 1 or 3 months depending on treatment received), and (3) a double blind (DB) phase (12 months). The 12-month DB phase included a total of 4 injections at 3-month intervals. The PK steady-state was assumed during the DB phase [10].

The mean PK data used in the analysis were digitized from the reference papers at different doses: ER single dose of 3 mg, 6 mg, 9 mg, 12 mg, and 15 mg; PP1M single dose of 25 mg, 50 mg, 100 mg, and 150 mg; PP3M single dose of 175 mg, 300 mg, 450 mg, and 525 mg and repeated doses of 350 mg, and 525 mg.

The data were analyzed using a non-linear mixed-effect modeling approach. In this model, a random effect (log-normally distributed) was used to account for the potential variability in the PK parameters associated with the different assessment of the mean PK time course used in the analysis (intra-measurements variability). The residual error model was assumed proportional the PK measurements. The analyses were conducted in NONMEM version 7.4 (ICON Development Solutions, Dublin, Ireland) using the ADVAN13 subroutine and the first-order conditional estimation with interaction method.

### Traditional modeling approach

**Fig. 1:**
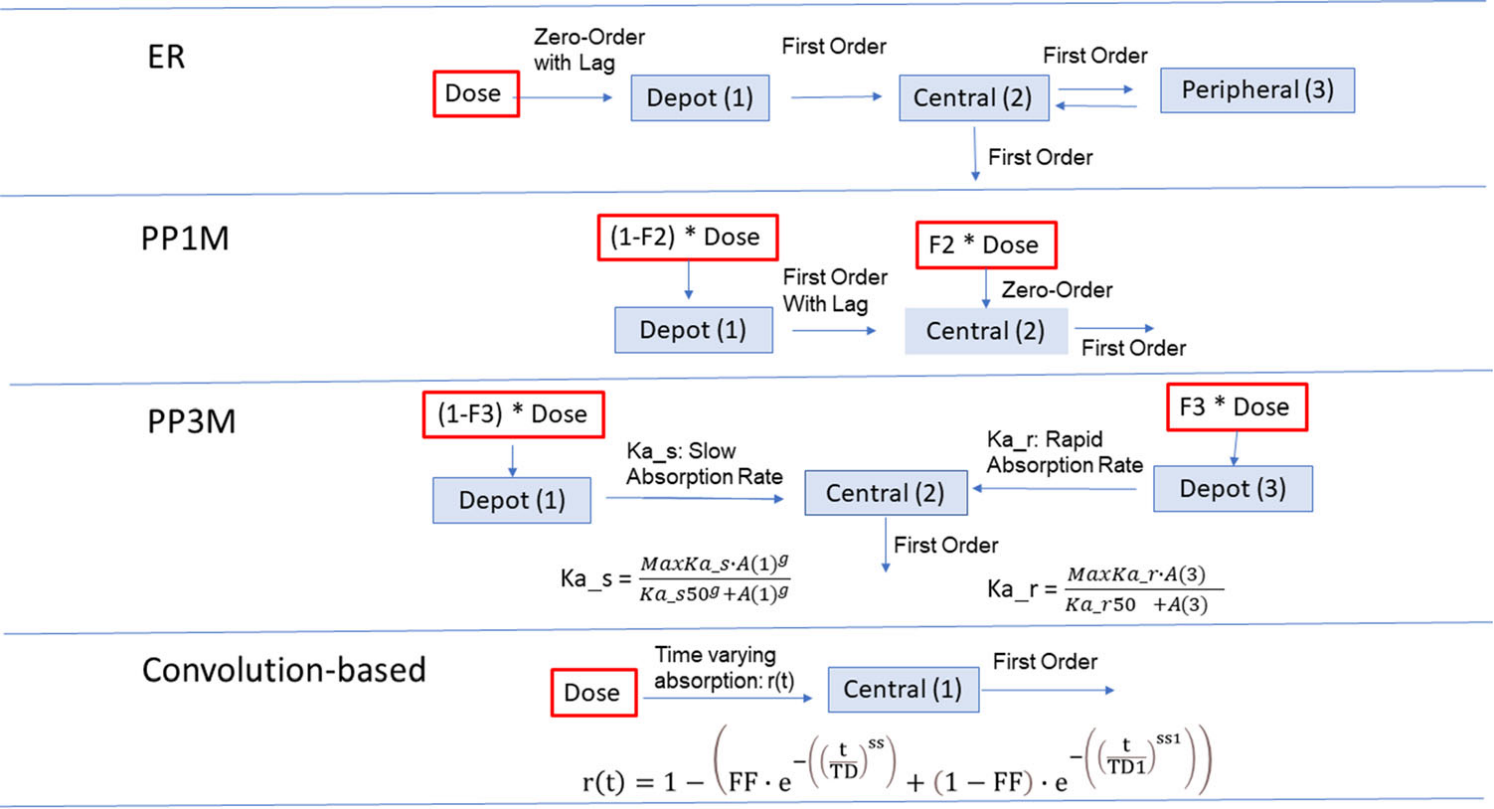
Schematic representations of the traditional and convolution-base models used in the analysis

The three different structural models initially used to implement the traditional approach (Fig. 1) were:

- *ER* two-compartment model (three differential equations) with consecutive zero- and first-order absorption with lag-time, and first-order elimination [11].
- *PP1M* one-compartment model (two differential equations) with first-order elimination and absorption described by a fraction of the dose released with zero-order process and a fraction with first-order process [12].
- *PP3M* The PP3M formulation had a considerably slower absorption profile than the PP1M formulation. Attempts to fit the PP1M model to PP3M data resulted in inadequate model performance. The final absorption sub model (three differential equations) described the absorption as two parallel saturable processes; one fast and one slow. The processes were parameterized using the Hill function, the slow absorption process included a Hill coefficient that was greater than 1 [13, 14].

### Convolution-based modeling approach

The time-course of the drug concentration resulting from an arbitrary dose can be described as a function of the in vivo drug release and the disposition/elimination processes defined by the unit impulse response accordingly to the convolution integral:

1 $$\text{C}\text{p}\left(\text{t}\right)={\int }_{0}^{\text{t}}\text{f}\left({\uptau }\right) \times \text{U}\text{I}\text{R}(\text{t}-{\uptau })\times \text{d}{\uptau }$$

where τ is a dummy variable used for integration, Cp is the plasma concentration as a function of time t, f is the drug input rate, and UIR is unit impulse response function.

The function characterizing the drug delivery f can be estimated as the first-derivative of the cumulative drug release function r:

2 $$\text{f}\left(\text{t}\right)=\frac{\text{d}\text{r}\left(\text{t}\right)}{\text{d}\text{t}}$$

The convolution integral model (Eq. 1) can be represented in a more manageable form using a system of differential equations. In case of simple disposition process (say one compartment with first order process), the UIR function is characterized by the volume of distribution (V) and by the first order elimination rate constant (kel). The equation describing Cp(t) (one differential equation) is:

3 $$\frac{\text{d}\text{Ap}\left(\text{t}\right)}{\text{dt}}=\text{F}\times \text{Dose}\times \frac{\text{dr}\left(\text{t}\right)}{\text{dt}}-\text{kel}\times\text{Ap}$$

4 $$\mathrm{Kel}=\frac{\mathrm{CL}}{\mathrm V},\;\mathrm{Cp}\left(\mathrm t\right)=\frac{\mathrm{Ap}\left(\mathrm t\right)}{\mathrm V}$$

where Ap(t) is the amount of drug, and F is the relative bioavailability of the current formulation with respect to the reference formulation (the one that provided an estimate of the UIR function defined by CL and V). In this scenario, Cp can be analytically estimated by numerically integrating Eq. 3. This model can easily be generalized to account for complex disposition processes including non-linearity in the PK distribution and elimination processes.

The implementation of the convolution-based model requires that one specify the sub-model characterizing the r(t) function. The structural form of r(t) was assumed to be described by a parametric function, such as: exponential or single and dual Weibull functions with unknown parameters. In case of a double Weibull function, the r(t) function can be written as:

5 $${\text{r}}\left( {\text{t}} \right) = 1 - \left( {{\text{FF}} \times {\text{e}}^{{ - \left( {\left( {\frac{{\text{t}}}{{{\text{TD}}}}} \right)^{{{\text{ss}}}} } \right)}} + \left( {1 - {\text{FF}}} \right) \times {\text{e}}^{{ - \left( {\left( {\frac{{\text{t}}}{{{\text{TD}}1}}} \right)^{{{\text{ss}}1}} } \right)}} } \right)$$

where t = time, FF = fraction of the dose released in the 1st process, TD and TD1 = times to release 63.2% of the dose in the 1st and in the 2nd process, and SS and SS1 = sigmoidicity factors for the 1st and the 2nd process, respectively. The dr/dt function can be analytically estimated using the first derivative of the Eq. 5 or can be approximated using a finite difference approach:

6 $$\frac{{{\text{dr}}}}{{{\text{dt}}}} \cong \frac{{{\text{r}}\left( {{\text{t}} - \Delta } \right) - {\text{r}}\left( {{\text{t}} + \Delta } \right)}}{{2 \times \Delta }}$$

where Δ is a sufficiently small number.

The schematic representations of the traditional and convolution-base models are presented in Fig. 1.

### Comparison of the models’ performances

The same single dose datasets for ER and LAI products were analyzed using the traditional and convolution-based modeling approaches. The Akaike (AIC) and Bayesian (BIC) information criteria were used for comparing the performances of the two modeling approaches. Among two models, the most informative will be the one with the lowest AIC and BIC values. In addition, the overall ability of the two modeling approaches to fit the data was evaluated using the average percent prediction error defined as 100 × [observations − predictions]/observations (%PE).

### Software

The data used in the analyses were extracted from the different referred publications using ScanIt plot digitizer software, version 2.0 [15]. The analyses were conducted using NONMEM, version 7.4 (ICON Development Solutions, Hanover, MD, USA). Graphical data presentations were conducted using R (R Foundation for Statistical Computing).

## Results

The paliperidone concentrations of each formulation (i.e., ER, PP1M, and PP3M), resulting from the administration of the different doses were jointly analyzed using a non-linear mixed effect approach using the traditional and the convolution-based models.

The estimated parameter values using the convolution-based model with the relative standard error for the ER, PP1M, and PP3M products are presented in Table 1.

**Table 1:** Estimated parameters using the convolution-based model with the relative standard error (RSE) for the ER, PP1M, and PP3M formulations

Parameter	ER	PP1M	PP3M
	Estimate (RSE)	Estimate (RSE)	Estimate (RSE)
TD (h)	0.864 (0.70%)	9.45 (12.90%)	12.6 (0.10%)
SS^a^	8.71 (1.20%)	3.14 (10.90%)	1.66 (0.30%)
TD1 (h)	1.54 (0.80%)	3.61 (27.70%)	6.32 (0.10%)
SS1^a^	0.395 (1.90%)	2.68 (12.60%)	2.16 (0.10%)
FF (%)	0.309 (3.20%)	0.355 (14.10%)	0.676 (0.10%)
CL/F (L/h)	15.13 (1.50%)	5.04 (9.70%)	4.38 (0.10%)
V/F (L)	446 (2.10%)	6080 (16.90%)	11,700 (0.20%)

*RSE* relative standard error

^a^Unitless

The estimated parameter values using the traditional modeling approach with the relative standard error for the ER, PP1M, and PP3M products are presented in the Supplementary Material 2.

**Fig. 2:**
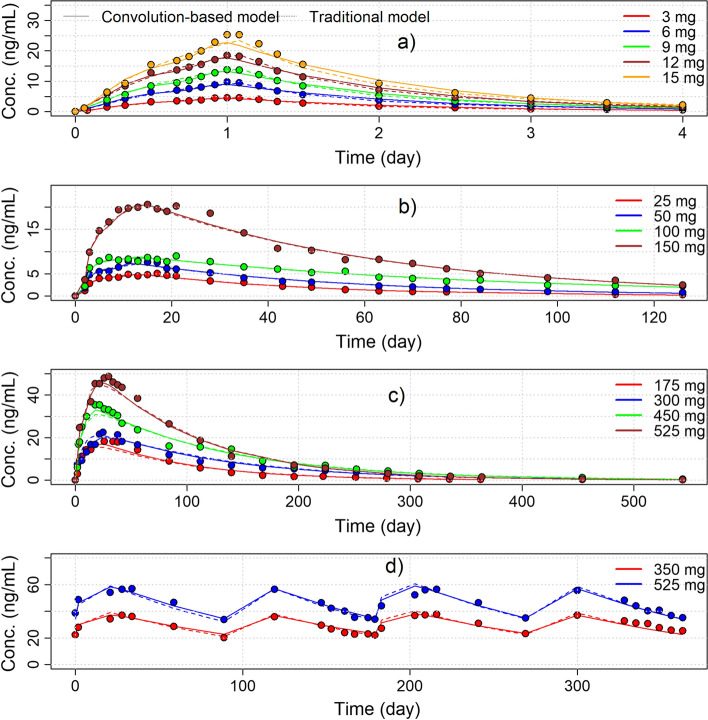
Mean observed data (dots) versus time with the model predicted concentrations estimated with the traditional (dotted lines) and the convolution-based (solid lines) approaches. Panel **a** ER formulation, panel **b** PP1M, panel **c** PP1M single dose, panel **d** PP3M repeated doses

The plots of observed and model predicted concentrations versus time estimated with the two modeling approaches are presented in Fig. 2. The comparison of the observed and model predicted concentrations indicated a good and comparable ability of the two-modeling approached for describing the mean concentrations of the 3 products at the different doses evaluated.

The summary results of the analyses with the comparison of the performances of the two modeling approaches are presented in Table 2. The results confirmed comparable performances with a numerical preference for the convolution-based model.

**Table 2:** Summary results of the analysis with the comparison of the performances of the two modeling approaches

Formulation	Method	AIC	BIC	Mean %PE (95%CI)
ER	Traditional	− 157.593	− 134.90	− 0.38 (− 1.70, 0.95)
	Conv-based	− 153.935	− 126.20	− 0.53 (− 2.23, 1.16)
PP1M	Traditional	− 106.54	− 78.68	− 0.50 (− 2.16, 1.16)
	Conv-based	− 107.66	− 77.27	− 0.49 (− 2.13, 1.14)
PP3M	Traditional	358.904	392.09	− 2.46 (− 5.40, 0.48)
	Conv-based	241.699	277.81	− 1.84 (− 4.77, 1.09)

*AIC* Akaike information criterion, *BIC* Bayesian information criterion, %*PE* % prediction error, 95%*CI* 95% confidence interval

The NONMEM code and data for the joint fitting of the single and repeated doses for PP3M is provided in the Supplementary Material 1.

**Fig. 3:**
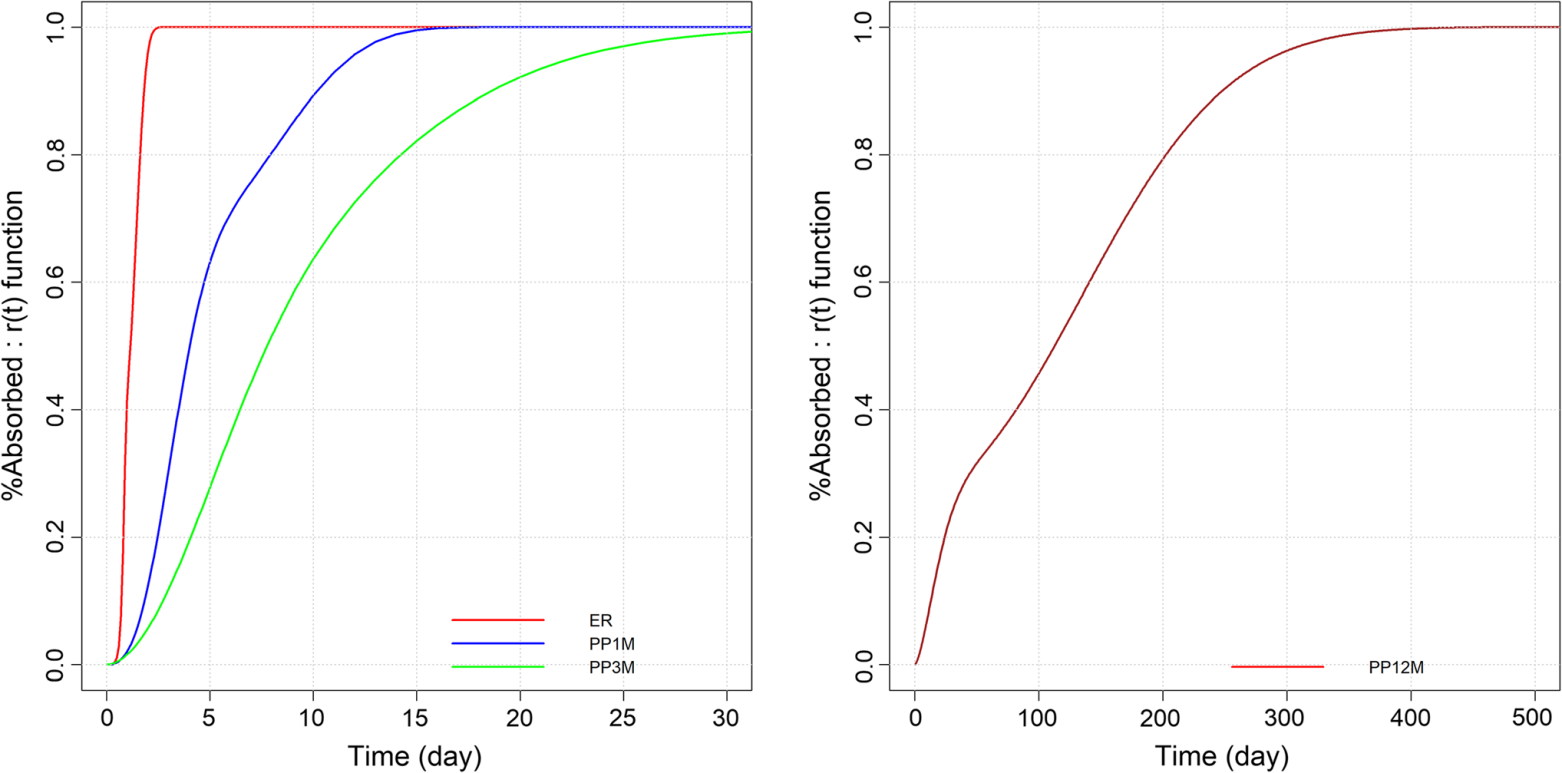
Left panel: fraction of the dose released in vivo computed using the double Weibull parameter values estimated in the modeling of the ER, PP1M, and PP3M products. Right panel: estimated fraction of the dose released in vivo associated with a hypothetical once-a-year (PP12M) LAI product

The fraction of the dose released in vivo computed using the double Weibull parameter values estimated in the modeling of the ER, PP1M, and PP3M products (Table 1) are presented in Fig. 3 (left panel).

The feasibility and the characteristics of a possible new paliperidone LAI once-a-year product were explored using the in vivo drug release function (i.e., the r(t) model defined by the Eq. 5).

The steady state paliperidone concentrations of the PP3M product estimated over a treatment time of 3 years were considered as the reference target exposure. The same disposition and elimination parameter values estimated in the PP3M analysis were used in the simulation. Alternative modified time varying input functions for PP12M were explored. The final values retained for the potential in vivo drug release of the PP12M products were: TD = 22 (h), SS = 1.6, TD1 = 180 (h), SS1 = 2.13, and FF = 0.2768 (%) (Fig. 3, right panel).

**Fig. 4:**
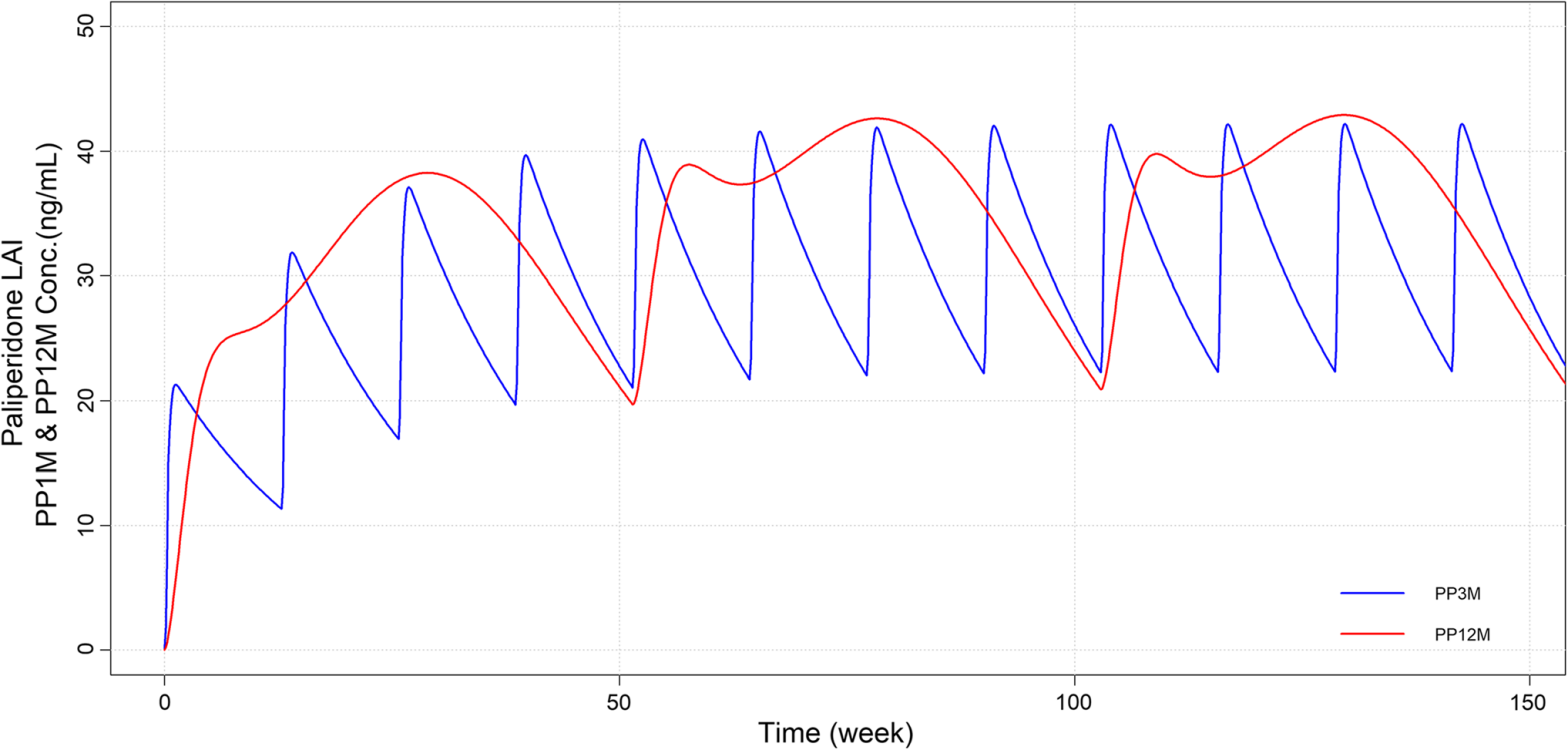
Comparison of the paliperidone plasma concentrations versus time for the reference PP3M product at the dose of 350 mg (blue solid line) and for the estimated PP12M at the dose of 1600 mg (red solid line) administered over 3 years

The plot of the paliperidone concentrations versus time at the dose of 350 mg for PP3M and 1600 mg for PP12M are presented in Fig. 4.

## Discussion

The aim of the analysis was to evaluate the features and the interest of a common integrated convolution-based structural model for describing the paliperidone PK resulting from the administration of extended-release oral dose, and once- and three- monthly LAI products.

In the present analysis the absorption rate was described by a double Weibull model.

Even though this model accurately described the data, this remains one among many possible models available for describing the complex absorption processes of LAI products. Among these models we can mention the transit compartment model, the sum of inverse gaussian models, and the combination of different drug release models such as parallel first-order absorption, mixed zero-order and first-order absorption, absorption window with or without Michaelis-Menten absorption [16–19].

The comparative performance of these modeling approaches was out of the scope of the present paper. Our objective was to compare the performances of traditional compartmental and convolution-based modeling approaches.

The results of the analyses indicated that the traditional and convolution-based models showed comparable performances in the characterization of the paliperidone PK. However, the convolution-based approach showed several appealing features that justify the choice of this modeling as a preferred tool for modeling LAI products.

In particular, this modeling approach: (a) can facilitate the development of IVIVC [20], (b) can be used to identify formulations with optimal in vivo release properties [6], (c) can be used for optimizing the clinical benefit of a treatment by supporting the implementation of integrated models connecting in vitro and in vivo drug release, in vivo drug release to PK, and PK to PD [21], and (d) the model is flexible enough to describe the paliperidone data previously characterized by three different models separately.

The mechanism of action of paliperidone is mainly associated with the antagonistic activity on the D2 receptors (a PD biomarker) at the level of the brain. It is currently widely accepted that a D2 occupancy ranging from 60 to 80% is needed for anti-psychotic’s clinical efficacy [22]. Based on data from a positron emission tomography (PET) scan study, a relationship between paliperidone plasma concentrations and D2 receptor occupancy was established, and plasma concentrations needed to achieve an effect on schizophrenic symptoms were well defined. An Emax model was developed and the paliperidone concentration associated with the 50% of the maximal response was estimated at 4.9 ± 0.53 ng/mL [23]. The relationship between paliperidone PK and D2 receptor occupancy is instrumental for determining the PK levels and the in vivo release rate appropriate for achieving the effective level of D2 occupancy over time.

A methodology for optimizing the clinical benefit of an antipsychotic treatment (defined as the ability to reach and maintain a D2 receptor occupancy ranging from 60 to 80%) was proposed using a nonlinear optimization algorithm operating on an integrated convolution-based drug-disease model [21]. In this analysis the clinical benefit was expressed as a nonlinear function of the in vivo drug release and dosage regimen. The results of the analyses indicated that a substantial improvement in clinical benefit can be obtained when optimal strategies for in vivo drug release and dose finding are deployed.

On these bases, the convolution-based modeling can be considered as a modeling framework facilitating the development of new LAI products with an improved benefit-risk ratio over different time intervals.

A case study is presented to illustrate how the convolution-based model developed using PP1M and PP3M products can be used to inform the development of alternative LAI products with even longer dosing interval such as a paliperidone LAI product administered once-a-year (PP12M).

The simulated plasma concentrations indicated that a similar steady state exposure of PP3M can be achieved with a repeated administration of PP12M once the in vivo drug release was characterized by a specific function. The knowledge of the shape of this function in conjunction with the availability of IVIVC can be utilized as tool for guiding the in vitro development of new formulations satisfying the target drug release properties.

These results provide a proof-of-principle of the feasibility of a once-a-year LAI product. Obviously, additional clinical and practical criteria should be considered for defining the optimal shape of the paliperidone exposure for such a long treatment time period.

The limitations of the methodology remain associated with the characterization of the UIR function required for implementing the convolution-modeling if the objective of the model would have been to conduct a deconvolution analysis, the use of the parameters for the UIR function estimated using IV data would have been mandatory. In this case, these parameters of the UIR function would have been fixed in the fitting procedure for the deconvolution analysis. In the context of the present analysis and in absence of IV data, the objective of the modeling was not to conduct a deconvolution analysis but simply to estimate the PK parameters (including CL/F and V/F) that best describe the data.

In the present analysis the values of the UIR function was empirically estimated for each product (ER, PP1M, and PP3M) using the available measurements resulting from LAI products treatment.

## Conclusion

In conclusion, the proposed modeling and simulation approaches have been shown to represent an effective framework for describing complex and multiphase PK of LAI products, for identifying the optimal dosing strategy, for facilitating the development of LAI formulations, and for deploying an effective Model-informed drug development (MIDD) process.

## Supplementary Information

Below is the link to the electronic supplementary material.

Supplementary file 1 (DOCX 18.2 kb)

Supplementary file 2 (DOCX 14.9 kb)
