# Supplementary material for: Convolution-based approach for modeling the paliperidone extended release and Long-Acting Injectable (LAI) PK of once-, and three-monthly products administration and for optimizing the development of new LAI products
Source: J Pharmacokinet Pharmacodyn. 2022 Dec 9;50(2):89–96. doi: 10.1007/s10928-022-09835-7 (PMC10066107; doi:10.1007/s10928-022-09835-7)
Supplement: Supplementary file 1 — (DOCX 18.2 kb) [file 10928_2022_9835_MOESM1_ESM.docx]

Convolution-Based Approach for modeling the Paliperidone Extended Release and Long-Acting Injectable (LAI) PK of Once-, and Three-Monthly Products Administration and for Optimizing the Development of New LAI products

NONMEM code and data for the joint fitting of the single and repeated doses for PP3M

$PROBLEM pp3m

$INPUT MM ID DV DOX TIME

;

; MM = 30 single dose

; MM = 3 repeated doses

;

$DATA ..\pp3m_pk.csv IGNORE=@

; Fragment of the input data file

;MM ID DV DOSE TIME

;3 350 0 350 0

;3 350 22.2671 350 361

;3 350 27.8522 350 363

;3 350 34.2436 350 382

;3 350 37.0019 350 389

;.. .. .. .. ..

;.. .. .. .. ..

;30 1 0 175 0

;30 1 2.992 175 2

;30 1 9.119 175 4

;30 1 11.22 175 6

;.. .. .. .. ..

;.. .. .. .. ..

;

$SUBS ADVAN13 TOL=6

$MODEL COMP(A)

$abbr declare dosetime(10),dose(10)

$abbr declare dowhile I

$abbr declare dowhile ndose

$abbr declare INPT

$PK

NNID=IREP

if(mm.eq.30) then

ndose=1

dosetime(1)=0

dose(1)=dox*1000

endif

if(mm.eq.3) then

ndose=8

dosetime(1)=0

dosetime(2)=30*3

dosetime(3)=30*6

dosetime(4)=30*9

dosetime(5)=30*12

dosetime(6)=30*15

dosetime(7)=30*18

dosetime(8)=30*21

dose(1)=dox*1000

dose(2)=dox*1000

dose(3)=dox*1000

dose(4)=dox*1000

dose(5)=dox*1000

dose(6)=dox*1000

dose(7)=dox*1000

dose(8)=dox*1000

endif

TD=THETA(1)*EXP(ETA(1))

cl=THETA(2)*EXP(ETA(2))

S1=THETA(3)*EXP(ETA(3))

SS=THETA(4)*EXP(ETA(4))

TD1=THETA(6)*EXP(ETA(5))

SS1=THETA(7)*EXP(ETA(6))

FF=THETA(8)*EXP(ETA(7))

DELT=0.001

kel=cl/s1

$DES

INPT=0

I=1

DOWHILE (I<=NDOSE)

TT=T-dosetime(I)

IF(TT>=0) then

TT1=TT

TT2=TT+DELT

ABS1=(FF*EXP(-(TT1/TD)**SS)+(1-FF)*EXP(-(TT1/TD1)**SS1))

ABS2=(FF*EXP(-(TT2/TD)**SS)+(1-FF)*EXP(-(TT2/TD1)**SS1))

KAB=(ABS1-ABS2)/DELT

INPT=INPT+dose(I)*KAB

endif

I=I+1

ENDDO

KKK=INPT

DADT(1)=KKK-KEL*A(1)

$ERROR

FFF=A(1)/S1

IPRED=FFF

ERR1=THETA(5)

ERR0=THETA(9)

WW=ERR0**2+ERR1**2*IPRED*IPRED

w=0.0001

if(ww.gt.0) w = SQRT(ERR0**2+ERR1**2*IPRED*IPRED)

IRES = DV-IPRED

IWRES = IRES/W

Y = IPRED + W*EPS(1)

$THETA

(0, 12.7) ;TD

(0, 105) ;cl

(0, 11600) ;V

(0, 1.64) ;SS

(0, 0.129) ;eps 1

(0, 2.15) ;SS1

(0, 6.41) ;TD1

(0, 0.67,1) ;FF

(0.00001) FIX ;eps 2

$OMEGA

0.0599 ;TD

0.0106 ;cl

0.0592 ;S2

0 FIX ;SS

0.0135 ;TD1

0 FIX ;SS1

0 FIX ;FF

$SIGMA 1 FIX
