# Supplementary material for: Convolution-based approach for modeling the paliperidone extended release and Long-Acting Injectable (LAI) PK of once-, and three-monthly products administration and for optimizing the development of new LAI products
Source: J Pharmacokinet Pharmacodyn. 2022 Dec 9;50(2):89–96. doi: 10.1007/s10928-022-09835-7 (PMC10066107; doi:10.1007/s10928-022-09835-7)
Supplement: Supplementary file 2 — (DOCX 14.9 kb) [file 10928_2022_9835_MOESM2_ESM.docx]

Convolution-Based Approach for modeling the Paliperidone Extended Release and Long-Acting Injectable (LAI) PK of Once-, and Three-Monthly Products Administration and for Optimizing the Development of New LAI products

Table 1s. Estimated parameters using the traditional modeling approach with the relative standard error (RSE) for the ER, PP1M, and PP3M formulations

| **ER** | | **PP1M** | | **PP3M** | |
| --- | --- | --- | --- | --- | --- |
| **Parameter** | **Estimate (RSE)** | **Parameter** | **Estimate (RSE)** | **Parameter** | **Estimate (RSE)** |
| CL/F (L/h) | 15(1.70%) | CL/F (L/h) | 5.04(5.30%) | CL/F (L/h) | 4.09(5.40%) |
| V/F (L) | 319(1.20%) | V/F (L) | 6030(4.60%) | V/F (L) | 8900(28.10%) |
| D1(h) | 1.07(0.90%) | F2 (%) | 0.469(2.70%) | F3 (%) | 0.301(8.40%) |
| Lag (h) | 0.0294(0.50%) | D2 (h) | 13.5(0.60%) | Ka-s (h-1) | 154(28.30%) |
| k12 (h-1) | 0.581(5%) | Ka (h-1) | 1.19(3.70%) | Ka_s50 (h-1) | 0.328(6.50%) |
| k21 (h-1) | 1.27(8.20%) | Lag (h) | 1.78(0.70%) | G(*) | 2.02(2.30%) |
| ka (h-1) | 397(5.10%) |  |  | Ka_r (h-1) | 0.00935(62.70%) |
|  |  |  |  | Ka_r50 (h-1) | 0.0197(2.10%) |
| *RSE=relative standard error* | |  |  |  |  |
| **=Unitless* |  |  |  |  |  |
